# Ketogenic Diet and Epilepsy: What We Know So Far

**DOI:** 10.3389/fnins.2019.00005

**Published:** 2019-01-29

**Authors:** Isabella D’Andrea Meira, Tayla Taynan Romão, Henrique Jannuzzelli Pires do Prado, Lia Theophilo Krüger, Maria Elisa Paiva Pires, Priscila Oliveira da Conceição

**Affiliations:** ^1^Epilepsy Department, Paulo Niemeyer State Brain Institute, Rio de Janeiro, Brazil; ^2^Neurology Department, Federal Fluminense University, Rio de Janeiro, Brazil; ^3^Neurology Department, Rio de Janeiro State University, Rio de Janeiro, Brazil

**Keywords:** refractory epilepsy, ketogenic diet, modified Atkins diet, low glycemic index, diet therapy

## Abstract

The Ketogenic Diet (KD) is a modality of treatment used since the 1920s as a treatment for intractable epilepsy. It has been proposed as a dietary treatment that would produce similar benefits to fasting, which is already recorded in the Hippocratic collection. The KD has a high fat content (90%) and low protein and carbohydrate. Evidence shows that KD and its variants are a good alternative for non-surgical pharmacoresistant patients with epilepsy of any age, taking into account that the type of diet should be designed individually and that less-restrictive and more-palatable diets are usually better options for adults and adolescents. This review discusses the KD, including the possible mechanisms of action, applicability, side effects, and evidence for its efficacy, and for the more-palatable diets such as the Modified Atkins Diet (MAD) and the Low Glycemic Index Diet (LGID) in children and adults.

## Introduction

Epilepsy is a disabling and common neurological disease, which can be controlled successfully in most patients with one or more antiepileptic drugs. Approximately 30% of patients with epilepsy have refractory epilepsy, that is, have a failure of adequate trials of two tolerated, appropriately chosen and used antiepileptic drug schedules to achieve sustained relief of seizures (Picot et al., 2008; Kwan et al., 2009). Some of these patients are not surgery candidates, so it is necessary to search for alternative treatments for epilepsy such as palliative surgery, neuromodulation, and a ketogenic diet (KD).

The classic ketogenic diet (CKD) consists of a high-fat and low-protein and carbohydrate diet, with restricted calories and fluids. The diet mimics the fasting state, altering the metabolism to use fats as a primary fuel source; catabolism of fatty acids in the liver produces ketone bodies (KB), which induces urinary ketosis (Rho, 2017).

Recent studies have found a significantly positive outcome with the use of the KD for treatment of refractory epilepsy in children and adults (Barborka, 1928; Neal et al., 2008; Kverneland et al., 2015; Liu et al., 2018).

Regardless of the efficacy of the KD, most patients discontinue the diet because of its unpalatable and restrictive features. In the last 20 years, new variants of the KD diet have emerged, including the Modified Atkins diet (MAD), a low-glycemic-index diet, which although it has a high fat content, allows more protein and does not restrict calories and fluids. Several studies have shown that the new variants of the KD have a similar efficacy to the CKD (Kossoff et al., 2006; Tonekaboni et al., 2010; Coppola et al., 2011; Miranda et al., 2012; El-Rashidy et al., 2013). As presently understood, the KD is involved in multiple mechanisms responsible for biochemical alterations, including cellular substrates and mediators responsible for neuronal hyperexcitability. However, it is not yet known with certainty whether the success of the KD is due to a single or several mechanisms (Bough and Rho, 2007; Lutas and Yellen, 2013; Rho, 2017; Youngson et al., 2017).

Because epilepsy is a metabolic disease (Clanton et al., 2017), interest in studies of alterations of metabolism by anticonvulsants such as the KD has increased, as has their importance for the treatment of drug-resistant epilepsy. This contribution reviews the use and effects of the KD and its variants for the treatment of adults and children with intractable epilepsy.

## Ketogenic Diet Past to Present

Dietary treatments for diseases have probably been used for over 2000 years (Yuen and Sander, 2014). Fasting is the only therapeutic measure against epilepsy recorded in the Hippocratic collection. Two Parisian physicians, G Guelpa, and A Marie, recorded the first modern use of starvation as a treatment for epilepsy in 1911 (Wheless, 2008). The modern use of this form of therapy began in the early 1920s (Lima et al., 2014; Yuen and Sander, 2014), when Drs. Stanley Cobb and W.G. Lennox of Harvard at Harvard Medical School observed the effects of starvation as a treatment for epilepsy, noting that seizure improvement typically occurred after 2–3 days (Wheless, 2008). In the same period, Dr. Russel M. Wilder a physician at the Mayo Clinic in Minnesota, suggested that a specific diet could produce similar benefits to fasting, and proposed a diet that produced ketonemia. He studied a series of patients with epilepsy and demonstrated a result equivalent to fasting and that was maintained for a much longer period. This new concept of diet was designated the “KD.” Peterman, also at the Mayo Clinic, described a composition of the KD similar to that used today (Wilder, 1921).

In 1970, Robert C. Atkins developed a weight-loss diet that restricted the intake of carbohydrates (Sharma and Jain, 2014), and this diet was later evaluated for seizure treatment. The first patient was a 7-year-old girl with intractable epilepsy due to a left parietal cortical dysplasia, who used the Atkins diet for a week in order to acclimate to the CKD. After 3 days, her seizures stopped, and she remained seizure-free for 3 years with continued dietary treatment (Kossoff et al., 2013). In 2006, this diet was first formally referred to as the “MAD” to distinguish it from the Atkins diet (Kossoff et al., 2013). The MAD has three significant differences from the first version: the induction phase of limiting carbohydrates is maintained indefinitely; high-fat foods are not only allowed, but encouraged; and the primary goal of the diet is seizure control (Atkins, 2002; Sharma and Jain, 2014).

Nowadays, despite the new generation of anti-epileptic drugs, 35% of patients remain refractory. Interest in dietary therapy continues as a means of treatment for this group, even more with advances in knowledge regarding the association of gut microbiota and neurological diseases.

## Classic Ketogenic Diet

### What Is Classic KD?

The CKD is rich in lipids (90%) and low in carbohydrates and protein, in order to produce ketosis, and simulates a starvation state. It is a rigid diet, mathematically and individually calculated, and medically monitored (Armeno et al., 2014). It must also provide adequate vitamins and minerals. The shift in the energy metabolism from glycolytic energy production to energy generation through oxidative phosphorylation (fatty acid b-oxidation and ketone-body production) is part of the anticonvulsant mechanism of the KD (Bough, 2008; Liu et al., 2018). This is discussed in more detail in the section on the mechanism of action.

### Indication and Contraindications

Traditionally, the KD has been considered the gold standard for the treatment of metabolic diseases such as Glucose Transporter Protein 1 (GLUT-1) deficiency syndrome and Pyruvate Dehydrogenase Deficiency. At present, the KD has been consistently reported as more beneficial, with more than 70% patients showing positive responses, as opposed to the average 50% response in several conditions such as infantile spasms (Table 1). The KD has also been used in other conditions with less evidence, but possible benefits (Table 2) (Kossoff et al., 2018). Additionally, the KD is an important alternative treatment for patients with refractory epilepsy (Rho, 2017) that are not surgery candidates.

**Table 1:** Epilepsy syndromes and some conditions in which the KD therapies has been reported probable benefit^∗^.

Angelman syndrome
Complex 1 mitochondrial disorders
Dravet syndrome
Epilepsy with myoclonic–atonic seizures (Doose syndrome)
Glucose transporter protein 1 (Glut-1) deficiency syndrome (Glut1DS)
Febrile infection–related epilepsy syndrome (FIRES)
Formula-fed (solely) children or infants
Infantile spasms
Ohtahara syndrome
Pyruvate dehydrogenase deficiency (PDHD)
Super-refractory status epilepticus
Tuberous sclerosis complex


*^∗^Adapted from Kossoff et al. (2018). Optimal clinical management of children receiving dietary therapies for epilepsy: Updated recommendations of the International Ketogenic Diet Study Group (Kossoff et al., 2018).*

**Table 2:** Epilepsy syndromes and some conditions in which the KD therapies has been reported possible benefit (one case report or series)^∗^.

Adenylosuccinate lyase deficiency
CDKL5 encephalopathy
Childhood absence epilepsy
Cortical malformations
Epilepsy of infancy with migrating focal seizures
Epileptic encephalopathy with continuous spike-and-wave during sleep
Glycogenosis type V65 Juvenile myoclonic epilepsy
Lafora body disease
Landau-Kleffner syndrome
Lennox-Gastaut syndrome
Phosphofructokinase deficiency
Rett syndrome
Subacute sclerosing panencephalitis (SSPE)


*^∗^Adapted from Kossoff et al. (2018). Optimal clinical management of children receiving dietary therapies for epilepsy: Updated recommendations of the International Ketogenic Diet Study Group (Kossoff et al., 2018).*

Kossoff et al. (2018) proposed that dietary therapy should be considered earlier as an option for treatment of intractable epilepsy, because of its proven efficacy, the poor chance of improvement with further anticonvulsant administration, and the possibility of using the MAD (Kossoff et al., 2006) and low-glycemic-index treatment (LGIT) (Pfeifer and Thiele, 2005), which are easier to manage in adults.

In contrast, some pathologies are considered contra-indicated for KD. Absolute contraindications have been described and summarized by Kossoff et al. (2018) (Table 3). The surgical epilepsies, whenever the patient or caregivers are having difficulty maintaining compliance with the diet, are relative contra-indications for KD (Table 4) (Kossoff et al., 2018).

**Table 3:** Absolute contraindications for the use of KD therapies^∗^.

Carnitine deficiency (primary)
Carnitine palmitoyltransferase (CPT) I or II deficiency
Carnitine translocase deficiency
β-oxidation defects
Medium-chain acyl dehydrogenase deficiency (MCAD)
Long-chain acyl dehydrogenase deficiency (LCAD)
Short-chain acyl dehydrogenase deficiency (SCAD)
Long-chain 3-hydroxyacyl-CoA deficiency
Medium-chain 3-hydroxyacyl-CoA deficiency
Pyruvate carboxylase deficiency
Porphyria


*^∗^Adapted from Kossoff et al. (2018). Optimal clinical management of children receiving dietary therapies for epilepsy: Updated recommendations of the International Ketogenic Diet Study Group (Kossoff et al., 2018).*

**Table 4:** Relative contraindications for the use of KD therapies^∗^.

Inability to maintain adequate nutrition
Surgical focus identified by neuroimaging and video-EEG monitoring
Parent or caregiver noncompliance
Propofol concurrent use (risk of propofol infusion syndrome may be higher)


*^∗^Adapted from Kossoff et al. (2018). Optimal clinical management of children receiving dietary therapies for epilepsy: Updated recommendations of the International Ketogenic Diet Study Group (Kossoff et al., 2018).*

### Pre-KD Counseling and Evaluation

To obtain the optimum engagement of the family and the patients, providing information and training is essential because the diet is difficult to maintain. Counselors should talk with the family about their expectations and make clear the efficacy rate and adverse events (AE), to reduce the abandonment of the diet. Websites, videos and publications, especially from support groups, can be very helpful and should be encouraged. It is also important to review the medications and change from oral solutions (carbohydrate content) to tablets (Armeno et al., 2014). The KD counseling, evaluation and follow-up should be done by a multidisciplinary team. A pediatric neurologist or neurologist and a nutritionist are the minimum team requirements.

Before starting the diet, the patient should maintain a seizure diary to establish a frequency parameter. Also needed are a laboratory evaluation including selenium and carnitine levels (Table 5), electroencephalogram (EEG), and a magnetic resonance image (MRI) of the brain. A renal ultrasound should be done in case of kidney stones; an electrocardiogram and carotid ultrasound are considered optional (Kossoff et al., 2018). The nutritional evaluation includes a nutritional anamnesis including a 3-day food report, food habits, allergies, aversions, and intolerances. Baseline weight, height, and the ideal weight for stature and body mass index (BMI) are needed to calculate the ketogenic ratio, calories, and fluid intake. The diet formulation should be established according to the patient’s age and the administration route (Kossoff et al., 2009).

**Table 5:** Laboratory evaluation^∗^.

Complete blood count with platelets
Electrolytes to include serum bicarbonate, total protein, calcium, zinc, selenium, magnesium, and phosphate serum
Liver and kidney tests (including albumin, blood urea nitrogen and creatinine
Fasting lipid profile
Serum acylcarnitine profile
Urinalysis
Urine calcium and creatinine
Anticonvulsant drug levels ^a^
Urine organic acids ^b^
Serum amino acids ^b^
Vitamin D level


*^∗^Adapted from Kossoff et al. (2018). Optimal clinical management of children receiving dietary therapies for epilepsy: Updated recommendations of the International Ketogenic Diet Study Group (Kossoff et al., 2018). ^a^if applicable; ^b^if diagnosis unclear.*

### Diet Initiation

The goal is to reach a ratio of four portion of fat to one portion of protein plus carbohydrate, described as “4:1.” To achieve this level, one of two approaches, with or without fasting, may be used. In the former approach, the patient must be hospitalized for 12–48 h, or when ketones are present in the urine (Rubenstein, 2008), to prevent the development of hypoglycemia and dehydration. This method tends to accelerate the development of ketosis although it can generate more stress on the patient (Armeno et al., 2014). When ketosis is reached, the meals are calculated to maintain a constant KD ratio, while calories are added until full-calorie meals are tolerated (Kossoff et al., 2009). The latter approach requires no hospitalization and the KD ratio increases weekly, from 1:1, 2:1 and 3:1 to 4:1 (Bergqvist et al., 2005). Most of the literature suggests that there is no significant difference between the two approaches in terms of the time needed to reach ketosis and the occurrence of hypoglycemia (Kim et al., 2004), so nowadays patients tend to not fast.

Taking into account that the KD provides only small amounts of fruits, vegetables, grains, milk and cheese, supplementation is essential. Low-carbohydrate multivitamin and mineral supplements should be taken daily.

### Follow Up

Patients on the KD should be seen regularly every 3 months, and the family should be able to easily contact the diet team to resolve possible doubts and discuss adverse effects. In each evaluation, the seizure dairy and the child’s cognitive development and behavior should be observed (Auvin and Nabbout, 2011). It has been noted that it is possible to improve the cognitive development and behavior even without a change in the seizure frequency. Although some authors have reported no relationship between the efficacy and the level of ketosis, it is still recommended to measure the urine ketosis several times a week (Kossoff et al., 2009).

For efficacy, the KD requires a period of at least 3 months from the time that the patient reaches ketosis, so it is important to encourage the patient and the family to continue with the diet for this period without changing the medication.

### Adverse Effects

Because KD is not a physiological diet, it is necessary to recognize and closely manage AE (Kossoff et al., 2009). Acute AE include dehydration, hypoglycemia, lethargy, metabolic acidosis, and gastrointestinal symptoms. However, most of the side effects involve weight loss, high levels of low-density lipoprotein, and elevated total cholesterol (Liu et al., 2018). Other important AE are gastrointestinal symptoms, which include constipation, diarrhea, vomiting, and abdominal pain.

The family should also be informed about how to recognize the symptoms of hypoglycemia and be advised to administer a small amount of juice or other forms of dextrose (Kossoff et al., 2018). Nephrolithiasis may also develop, and an abdominal ultrasonography should be requested.

## Mechanism of Action

The understanding of the mechanisms of action of KD is incomplete; however, some theories have been advanced about how it modifies the neuronal metabolism and excitability in order to reduce the seizure frequency. Possibly, the real mechanism of reduction of cortical hyperexcitability involves multiple factors. Some of the systems involved in seizure reduction are related to metabolic changes in the blood and cerebrospinal fluid (CSF), including a decrease in glucose levels and an increase in KB. The mitochondria function and energy reserve may also play a role in the KD mechanisms, resulting in synapse stabilization and excitatory decrease.

### Ketone Bodies: Anticonvulsant Effects

Ketone bodies, acetoacetate, and β-hydroxybutyrate (βOHB), are byproducts of fatty acid oxidation in the mitochondrial matrix of the hepatocytes. There are many theories about the role of KB, but the existence of an anticonvulsant effect is controversial. Some authors have found no relationship between KB and synaptic transmission and seizure control.

Experimental studies in an animal model showed that in rats exposed to KD there was no change in synaptic plasticity, using paired-pulse modulation and long-term potentiation (Thio et al., 2010). Similarly, Likhodii et al. (2003) did not detect any anticonvulsant effects in either ketone body (Likhodii et al., 2003). In spontaneously epileptic *Kcna1*-null mice, KB supplementation resulted in attenuation of electrographic seizure-like events (Kim et al., 2015). These authors also observed an inhibitory effect of KB on mitochondrial permeability transition related to apoptotic and necrotic death. Moreover, in experimental models, acetoacetate exerted a broad-spectrum anticonvulsant effect (Rho et al., 2002). In another study, Rho (2017) described a relationship among KB, neurotransmitter release and ATP-sensitive potassium channels (Rho, 2017). Similarly, to these studies, injection of KB led to the reduction of seizure susceptibility (Gasior et al., 2008). Ma et al. (2007) found a decrease of the spontaneous firing rate in sections of mouse tissue, which was eliminated in the absence of ATP-sensitive potassium channels (KATP). In addition, KB can exert a direct inhibitory effect on the vesicular glutamate transport (Juge et al., 2010). It is possible that these divergent results are related to the different concentrations of KB used in these studies and the diverse seizure thresholds of the animal models. These conflicting results can be also explained by differences in diet composition.

### Neuronal Metabolism and Synaptic Function

Another hypothesis regarding the function of the KD is related to changes in neuronal metabolism, mitochondrial function and energy reserve, and the environment. In normal conditions, the usual substrate for the neurons is glucose. To facilitate its diffusion through the brain-blood barrier, glucose transports are present in the brain capillary endothelial layer (Greene et al., 2003). The glucose metabolism produces the rapidly available energy that is necessary for seizure activity. Therefore, in patients on the KD, the blood glucose energy levels are low, and the brain begins to use KB for energy. This anaerobic metabolism slows the energy availability, which reduces seizures. The anticonvulsant propriety of a decrease in glucose metabolism has been shown in experimental models in which the administration of 2-Deoxy-D-glucose elevates the seizure threshold (Garriga-Canut et al., 2006). The anticonvulsant effect of the KD can be quickly reversed after glucose infusion (Huttenlocher, 1976). Based on these data, we can postulate the influences not only of the KB, as discussed above, but also the reduction in glucose levels as a mechanism of action of the KD.

Chronic ketosis may play a role in the KD anticonvulsant properties, since it has been shown that chronic ketosis elevates the brain energy reserve via stabilization and reduction of excitability of synapses (Devivo et al., 1978). The energy reserve is directly associated with mitochondria, which is an important element to consider in the antiepileptic effect of KD. Bough et al. (2006) demonstrated an increase in mitochondria biogenesis in an experimental model of rats fed with KD, indicating an increase in the energy stores (Bough et al., 2006). The increase in mitochondrial metabolism leads to an increase in ATP production, which activates KATP, in turn attenuating neuronal excitability. This activation may be associated with adenosine A1 receptors (Li et al., 2010) and GABAB receptors (Mironov and Richter, 2000).

In this process, we can postulate that modifications of the metabolism are associated with an increase of ATP, and improve mitochondrial capacity and cell energy, with an increase in metabolic resilience.

### Neurotransmitter Function

The KD-induced synaptic stabilization is additionally related to changes in critical amino acids as a result of ketone metabolism. It has been proposed that KD interferes with the concentration of gamma-aminobutyric acid (GABA), the major inhibitory neurotransmitter. There is evidence in clinical practice of increased GABA levels in the CSF of patients on the KD diet (Wang et al., 2003). The decrease in aspartate levels promoted by KB lead to the synthesis of GABA. This occurs because of the inhibitory effect of aspartate on glutamate decarboxylase and the facilitation of the conversation of glutamate to glutamine in the astrocytes (Yudkoff et al., 2008). Not only can GABA be increased, but also other neurotransmitters such as adenosine A1 can be implicated in the anti-seizure effect of the KD (Szot et al., 2001). However, more evidence is needed.

### Gut Microbiota, Inflammation, and Genetic

The role of gut microbiota has recently been studied for its effect on several diseases, especially those with some inflammatory involvement. Several metabolic pathways are known to be modulated by the gut microbiota. Olson et al. (2018) demonstrated the impact of gut microbiota on the anti-seizure effect of KD. She found that KD modifies the gut microbiota, with a decrease in alpha-diversity and increases in the putatively beneficial bacteria *Akkermansia muciniphila* and *Parabacteroides* spp. This microbiota transformation leads to changes in the colonic luminal metabolome, with a decrease in gamma-glutamyl amino acids. This increases the GABA/glutamate content in the brain by decreasing gamma-glutamyl amino acids in the blood (Olson et al., 2018). In an acute electroshock model, it is reported that KD confers protection against seizures. Moreover, KD decreases the frequency of spontaneous seizures in Kcna1 knockout mice (Kim et al., 2015). In summary, changes in the gut microbiota seem to be important for the KD-mediated seizure protection.

The role of inflammatory cytokines in epilepsy is well known, and there is evidence that KD also interferes with pro-inflammatory cytokines. Dupuis et al. (2015) showed a peripheral and brain reduction of interleukin 1β and other pro-inflammatory cytokines in rats treated with KD in the LPS model.

Notably, there is a relationship between metabolic and epigenetic modifications. Shimazu et al. (2013) observed that βOHB inhibits class I histone deacetylases. During the KD, the elevation of βOHB causes changes in large-scale gene transcription but particularly those linked to oxidative-stress resistance factors. This result emphasizes that the KD has a potential role as a disease-modifying treatment in epilepsy.

In conclusion, all the mechanisms described above lead to systemic modifications and a dynamic metabolic homeostasis, in which the interplay among KB, glucose levels, mitochondrial function, synaptic neurotransmitters, and channel modifications can lead to changes in the seizure threshold and hyperexcitability. These changes contribute to the final antiseizure mechanism of KD.

Multiple mechanisms of action may explain why the modification of the KD can be effective even without ketosis. Importantly, the KD systemic action can have a broad spectrum of effects that may be beneficial in the treatment of different types of epilepsy and associated comorbidities such as cognition impairment, psychiatric disturbance, and sudden unexplained death.

## Modified Atkins Diet in Patients With Refractory Epilepsy

### Definition and Diet Composition

The MAD aims to provide increased flexibility and palatability, with a 1:1 ratio of fat to carbohydrates and protein, and contains around 65% fat, 25% protein, and 10% carbohydrate (Payne et al., 2018). Fat is encouraged and the carbohydrate intake is limited to 10–20 g/day in children and 15–20 g/day in adults (Kossoff, 2004; Kossoff and Dorward, 2008). Because of carbohydrate restriction, the MAD can also produce urinary ketones (Carrette et al., 2008). The MAD does not require weighing food on a gram scale, or restriction of calories, protein or liquids, and may be a good option for patients who are unable to tolerate a more restrictive diet such as the classical ketogenic diet (KD) (Cervenka et al., 2012). Low-carbohydrate multivitamin and calcium carbonate supplementation is recommended in the MAD (Kossoff et al., 2009).

### Efficacy in Children

Several studies have shown that the MAD, besides being more palatable, is as effective as the KD in the treatment of drug-resistant epilepsy in children (Miranda et al., 2011; Martin et al., 2016). A study performed using 20 children receiving 10 g of carbohydrates daily showed that 65% of the children had a >50% seizure reduction, 35% of the children had >90% improvement, and four children were seizure-free at 6 months (Kossoff et al., 2006). In a study in South Korea, 36% of 14 children treated with the MAD showed improvement of >50% in seizures and 12% were seizure-free (Kang et al., 2007). A recent meta-analysis performed using 70 studies concluded that the MAD and classical KD do not differ in reduction of seizure frequency at month 3 and month 6, with ≥50% and ≥90% reductions, respectively (Rezaei et al., 2017). A retrospective study showed >50% of seizure reduction in 65% of the 10 children who remained on the diet for up to 6 months, and 20% of them were seizure-free (Park et al., 2018).

Treatment with MAD was shown to be more effective in seizure control when the MAD was started with lower carbohydrate limits (Kossoff et al., 2010). In a randomized study with 20 children with drug-resistant epilepsy, 60% of them showed fewer seizures in the first 3 months on the MAD, with 10 g/day of carbohydrate intake against 10% of reduction with 20 g/day (*p* = 0.03). In the same study, after 3 months, an increase in carbohydrate intake to 20 g/day, maintained seizure control and improved tolerability, suggesting that a lower carbohydrate limit is important only in the first 3 months (Kossoff et al., 2007; Kossoff and Dorward, 2008).

### Efficacy in Adolescents and Adults

The efficacy of the MAD is also proven for the treatment of drug-resistant epilepsy in adults and adolescents. In this patient group, carbohydrate intake is generally around 15–20 g/day and the rates of seizure reduction and adherence are lower compared to those of the child population (Kossoff et al., 2008; Zare et al., 2017; Payne et al., 2018).

In a recent meta-analysis, eight studies were identified that used the MAD in adult patients with refractory epilepsy, aged between 15 and 86 years, with treatment times ranging from 3 to 36 months. In these studies, the proportion of patients who showed >50% seizure reduction ranged from 20 to 70% and the rate of seizure freedom ranged from 7 to 30%. The rate of abandonment of the diet varied between 12.5 and 82% of the patients (Liu et al., 2018).

### Side Effects

The MAD has been shown to be better tolerated than the classical KD, but some typical side effects such as gastrointestinal complaints, dyslipidemia and weight loss are reported (Zare et al., 2017). Beneficial effects have also been reported, such as mood improvement (Carrette et al., 2008).

## Low Glycemic Index Diet in Patients With Refractory Epilepsy

### Definition and Diet Composition

The use of the LGIT in the treatment of drug-resistant epilepsy was initially reported in 2005 by Pfeifer and Thiele (2005). This alternative diet treatment is based on a ratio 0.6:1 of fat to carbohydrates and protein, containing 60% fats, 30% protein, and 10% carbohydrates with a low glycemic index (GI) (GI<50) (Pfeifer and Thiele, 2005; Payne et al., 2018). The GI measures the tendency of a food to raise the blood glucose levels, compared to an equivalent amount of the reference carbohydrate, usually glucose (Pfeifer et al., 2008). Compared to classic the KD, the LGIT produces a smaller increase in ketone body levels, but has comparable efficacy, better tolerability and easier implementation (Pfeifer and Thiele, 2005; Pfeifer et al., 2008).

### Efficacy

The LGIT has proven to be effective in the treatment of focal and generalized epilepsies, with a reduction in seizure frequency occurring at 3–14 months and seizure control continuing for at least 1 year after the end of treatment (Pfeifer et al., 2008; Kim et al., 2017; Rezaei et al., 2018). Pfeifer and Thiele (2005) reported the use of LGIT in 20 drug-resistant epilepsy patients aged 5 to 34 years. After an average of 20 weeks of treatment, 50% of the patients had a >90% reduction in seizures. Coppola et al. (2011) studied 15 children, adolescents and young adults with refractory epileptic encephalopathies treated with LGIT. After 12 months they found 75–90% seizure reduction in 6 patients (40%), 50% reduction in 2 patients (13.3%), and the seizure frequency unchanged in 7 (46.7%). In a retrospective review of LGIT in 76 children, Muzykewicz et al. (2009) found an overall >50% reduction in seizure frequency in 50% of the patients at 3 months, which reached 66% at 12 months.

However, according to a recent systematic review, the positive results for LGIT efficacy in epileptic patients are doubtful because of the low number of high-quality studies. In this review, which included all electronic literature databases until July 2017, the authors found only eight studies with good or fair quality (69).

### Side Effects

Constipation and vomiting are the most common adverse effects reported in patients on the LGIT (Rezaei et al., 2018).

## Conclusion

The CKD and its variants should be considered as an alternative for non-surgical pharmacoresistant patients with epilepsy, of any age. Each patient must have an individually designed diet; however, adult patients have more difficulty in maintaining the CKD. It is essential to inform the patient and the family about the efficacy and AE related to the KD, and the use of websites and videos may help in this education. Although several theories exist regarding the mechanisms of action, further study is needed nevertheless the positive results are probably due to several mechanisms.

## Author Contributions

All the authors contributed substantially to the writing and revising of the manuscript. ID’A, HP, TR, MP, PC, and LK participated in the conception and design of the study, collected the literature, prepared the tables, and wrote the manuscript. ID’A, TR, and HP reviewed and edited the manuscript and approved the final version.

## Conflict of Interest Statement

The authors declare that the research was conducted in the absence of any commercial or financial relationships that could be construed as a potential conflict of interest.
